# International migration and caesarean birth: a systematic review and meta-analysis

**DOI:** 10.1186/1471-2393-13-27

**Published:** 2013-01-30

**Authors:** Lisa Merry, Rhonda Small, Béatrice Blondel, Anita J Gagnon

**Affiliations:** 1 Ingram School of Nursing, McGill University, Montreal, QC, Canada; 2 Mother and Child Health Research, La Trobe University, Melbourne, VIC, Australia; 3 Unité 953, Recherche épidémiologique en santé périnatale et santé des femmes et des enfants, Institut National de la Santé et de la Recherche Médicale (INSERM), Paris, France; 4 Ingram School of Nursing and Department of Obstetrics and Gynecology, McGill University; McGill University Health Centre (MUHC), Montreal, QC, Canada

**Keywords:** Caesarean, Immigrants, Refugees, Risk factors, Meta-analysis

## Abstract

**Background:**

Perinatal health disparities including disparities in caesarean births have been observed between migrant and non-migrant women and some literature suggests that non-medical factors may be implicated. A systematic review was conducted to determine if migrants in Western industrialized countries consistently have different rates of caesarean than receiving-country-born women and to identify the reasons that explain these differences.

**Methods:**

Reports were identified by searching 12 literature databases (from inception to January 2012; no language limits) and the web, by bibliographic citation hand-searches and through key informants. Studies that compared caesarean rates between international migrants and non-migrants living in industrialized countries and that did not have a ‘fatal flaw’ according to the US Preventative Services Task Force criteria were included. Studies were summarized, analyzed descriptively and where possible, meta-analyzed.

**Results:**

Seventy-six studies met inclusion criteria. Caesarean rates between migrants and non-migrants differed in 69% of studies. Meta-analyses revealed consistently higher overall caesarean rates for Sub-Saharan African, Somali and South Asian women; higher emergency rates for North African/West Asian and Latin American women; and lower overall rates for Eastern European and Vietnamese women. Evidence to explain the consistently different rates was limited. Frequently postulated risk factors for caesarean included: language/communication barriers, low SES, poor maternal health, GDM/high BMI, feto-pelvic disproportion, and inadequate prenatal care. Suggested protective factors included: a healthy immigrant effect, preference for a vaginal birth, a healthier lifestyle, younger mothers and the use of fewer interventions during childbirth.

**Conclusion:**

Certain groups of international migrants consistently have different caesarean rates than receiving-country-born women. There is insufficient evidence to explain the observed differences.

## Background

The use of medical interventions in birth has quickly risen in the last quarter century particularly in industrialized countries where the development of medical technology has advanced rapidly [1]. Most notable has been the dramatic rise in caesarean births with rates in the last 15 years in the US, Canada, Australia and parts of Europe reaching 25% and above (see http://www.oecd.org/els/health-systems/oecdhealthdata2012-frequentlyrequesteddata.htm). While a caesarean birth can be a life-saving procedure it is associated with a significantly increased risk of maternal death from complications of anaesthesia, puerperal infection, and venous thromboembolism [2]. The World Health Organization therefore recommends that caesareans be performed only in medically necessary instances [3].

Migration flows are increasing globally [4] and migrants contribute substantially to the total number of births, exceeding in some Western countries one fifth of births [5,6]. The health of migrants, including perinatal health, has therefore become a research priority [7]. Migrants (immigrants, refugees, asylum-seekers, undocumented and others with temporary or irregular statuses) [8,9] face multiple and intersecting social determinants of health that may compound childbearing health risks, including lower social economic status, lack of support, high levels of stress related to migration and resettlement and barriers in accessing healthcare [10,11]. Refugees and asylum-seekers may also have suffered trauma and abuse [12,13]. Perinatal health disparities [14], including disparities in caesarean births have been observed between migrants and receiving-country-born women and some literature suggests that non-medical factors such as communication barriers, support and/or care practices during labour and delivery, female genital cutting, or cultural preference may also be implicated in caesarean rate differences [15-17]. We conducted a systematic review to address the questions: 1) Do international migrant women in Western industrialized countries have consistently different rates of caesarean birth than receiving-country-born women? And 2) What are the mechanisms (medical and/or non-medical factors) that might explain differences?

## Methods

The “Meta-analysis of Observational Studies in Epidemiology (MOOSE)” recommended guidelines for the publication of meta-analyses of observational studies were used to prepare this manuscript [18].

We searched for cross-sectional and cohort studies that provided comparisons of caesarean rates between migrant and non-migrant women. To be included in the review, studies must have examined migrants who crossed international borders and who were living in an industrialized country, defined using the Organization for Economic Cooperation and Development (OECD) member list; studies conducted in refugee camps or with internally displaced persons were excluded. No restrictions were applied based on migrant origin, status or length of time in receiving-country. We searched the following electronic citation databases: Medline, Health Star, Embase, PsycInfo, CINAHL, Sociological Abstracts, Web of Science, Proquest Research Library, Proquest Dissertations and Theses, POPLINE, Global Health and PAIS. The search strategy was developed in conjunction with a McGill University Health Sciences librarian and can be found in Additional file 1. No language limitations were applied. Searches were conducted from inception of each database until January 2012.

We also hand-searched reference lists of identified literature, conducted web searches and contacted migration and health research experts for additional literature. Web sites searched included professional agency [(e.g., International Federation of Gynecology and Obstetrics (FIGO)] and government (Canada, US and Australia) health sites (see Additional file 2). Each site was searched by browsing through relevant indices predefined by each website and by using ‘search boxes’. Terms included: “c(a)esarean, c-section, mode of delivery, (im)migrant, (im)migration, foreign, country of birth”. Requests for literature were made through ROAM (Reproductive Outcomes and Migration), an international research collaboration, involving over 30 researchers from 13 countries, including Canada, Australia and Europe (see http://migrationandreproductivehealth.org/?page_id=76).

We reviewed all abstracts (or full articles if necessary) to determine if inclusion criteria were met. Those included were subsequently fully reviewed and quality-assessed and data were extracted into a database in Excel^©^. Non-English language reports were reviewed by ROAM collaborators and if no researcher could read the language of the report, we reviewed the English version abstract only.

The US Preventative Services Task Force criteria were used to assess the quality of studies [19]. Studies were evaluated on the 1) comparability of the study groups (assembly and maintenance); 2) degree of participant loss; 3) clarity of their “migrant” and “non-migrant” classifications; 4) validity and reliability of the approaches used to measure the migrant, caesarean birth and other variables; and 5) attention given to potential confounding factors (i.e., age and parity). Studies with a “fatal flaw” (i.e., ≥ 50% of the original sample lost to analysis and/or comparison groups not adequately defined) were removed from further analysis. Studies were scored as either “good” or “fair”- the former if the study met all quality criteria and the latter if it did not meet all criteria but had no fatal flaw. One reviewer assessed all studies and a second reviewer independently assessed 25% of the studies to confirm the assigned quality scores. Discrepancies were resolved through discussion.

A standardized coding form was developed to extract three types of data: 1) publication year and language; 2) study characteristics (e.g., study sample and data sources) and 3) results. The migration indicator (i.e., how migrants were labelled in the study) was recorded verbatim from the report and categorized by type as: “source country/region”, “foreign-born/non-national”, “ethnicity”, “length of time” or “migration status”. Sources of data were classified as: 1) population-based registry; 2) population-based hospital data; 3) population-based survey; or 4) hospital records/research study. Timeframe and geographical coverage (local, regional, or national) of data collection, host country and number of migrants and non-migrants were also recorded. Extracted results included frequencies of caesarean rates (overall, emergency and elective) and indications for caesarean for migrants and non-migrants separately. Explanations for caesarean rate differences between groups postulated in discussion sections were also extracted.

For each study we defined caesarean rates for migrant women as being “higher”, “lower” or “not different” compared to non-migrant women. If a study had results reported for more than one migrant group comparison they were coded as: “higher” if migrant results in all comparison groups were higher or a combination of higher and not different; “lower” if all were lower or lower and not different; “mixed” if some were higher and some lower; and “not different” if all rates between the migrant and non-migrant groups were not statistically different.

We used Review Manager 5.1^©^ to perform meta-analyses to estimate the ‘effect’ of being a migrant on the outcomes of interest: overall caesarean and emergency and elective caesarean rates. The studies were assumed to be heterogeneous due to the range of host countries and migrant groups examined and changes in obstetrical practice over time. We therefore used a random effects model which regards an effect as variable across studies and produces more conservative effect estimates with wider confidence intervals [20]. We combined and analyzed migrant sub-groups defined using UN macro-regions (see Additional file 3) or country of origin, migration status, and length of time in receiving-country. Sub-analyses by parity, receiving- country/region and time period of data collection were also performed. Due to the diversity across studies in variables selected to control for confounding we chose to use unadjusted results in meta-analyses. We calculated each study’s effect size as an odds ratio (OR) using the Mantel-Haenszel model (event/total for migrants and receiving-country women respectively) [20]. Attention was given to ensure duplicate data from different reports were not included twice in analyses and only one comparison from each study was combined for meta-analyses to ensure the results from a comparison group were not counted more than once. A weighted summary effect was calculated for each analysis performed.

To investigate the robustness of results which showed migrant women to consistently have different rates of caesarean compared to receiving-country-born women, sensitivity analyses were performed by limiting the analyses to only population-based data. To assess whether results would differ when covariates were adjusted, we also performed analyses by using adjusted odds ratios and applying the generic inverse variance method to produce summary estimates. Heterogeneity for all meta-analyses was assessed using I^2^ tests and we considered a value of 50% or more to indicate substantial heterogeneity [20]. We also examined confidence intervals among the studies in each forest plot to assess the degree of overlap; less overlap being interpreted as evidence of heterogeneity [20]. Publication bias was assessed by visual examination of funnel plots of the OR against the standard error of the log of the OR of studies within each migrant subcategory [20].

## Results

The database search yielded over eight thousand citations (n=8565). Once duplicates and studies that did not meet the inclusion criteria were excluded, 87 reports remained. Seventeen additional reports were identified: seven through hand searches, nine from ROAM collaborators and one annual report published by the New South Wales (Australia) Department of Health [21] online. Three reports could not be reviewed because of language [22-24]. Twenty studies were removed because the comparison group included migrants, and/or the migrant group included native-born, and/or the assembly of the migrant and non-migrant groups were dissimilar. Seventy-six studies met the inclusion criteria and were assigned a quality score (see Table 1 for a complete description of included studies) [15-17,25-103]. Twenty-two studies were scored as good and 54 were scored as fair (see Table 2). The primary reasons for not meeting all of the quality criteria was a lack of attention to confounding and ambiguity on how study groups (migrants vs. non-migrants) were defined or confirmed.

**Table 1:** Description of included studies

**Reference (language of publication)**	**Method (geographical coverage, data source, data yr)**	**Receiving country**	**Population**	Findings Migrants vs. Non-migrants*(Overallcaesarean rates)(Emergency&/orElective caesarean rates)	**Quality**
Alonso CP, Maresca MI, Oritz TA, & Serrano MM. (2006) (Spanish)	Local, hospital records, 2000-2002	Spain (Madrid)	2759 African, American, European, & Oriental "immigrants" vs. 3990 Spanish women.	L & ND overall (not statistically tested)	Fair
Aurelius G, & Ryde-Blomqvist E. (1978) (English)	Local, hospital records, 1968-1969	Sweden (Stockholm)	1235 “Immigrants” from Scandinavia, West, South, & East Europe, & “other countries” vs. 412 Swedish women. Only singleton births included.	H & ND overall (unadjusted)	Fair
Barron SL, & Vessey MP. (1966) (English)	Local, hospital records, 1958-1960	UK (South London)	1563 West Indian & Irish “immigrants” vs. 3891 British women. Only singleton births.	H overall (not statistically tested; W Indian: primips H, multips ND; Irish: primips ND, multips H)	Fair
Berger C, Liska G, Gallier J, & Soutoul JH. (1973) (French)	Local, hospital records, 1970-1972	France (Tours)	800 Portuguese/Spanish, North African, Yugoslavia, “other” “migrants” vs. 2655 French women.	M overall (not statistically tested)	Fair
Berger C, Laugier J, & Soutoul JH. (1974) (French)					
Bona G, Zaffaroni M, Cataldo F, Sandri F, & Salvioli GP. (2001) (English)	National, population-based hospital data, 1996-1997	Italy	3347 “Legal immigrants from developing countries” (Latin America, North Africa, Sub Saharan Africa, Middle East, Indian Sub-continent, Far East, Oceania, Eastern Europe, & Gipsies) vs. 6694 Italians.	ND overall (all immigrants combined; unadjusted; Variation by groups but not statistically tested)	Fair
Braveman P, Egerter S, Edmonston F, & Verdon M. (1995) (English)	Regional, population-based registry, 1991	US (California)	81,445 Asian and Latina “foreign born” vs. 93,685 “Whites”. Only singleton primiparous women.	L & ND overall (adjusted)	Good
Cassell E. (1995) (English) *Thesis*	Regional, population-based registry, 1982-1992	Australia (Victoria)	5268 Filipino-born vs. 507,457 Australian-born.	H overall (stratified by parity and age caesarean rates consistently H) H emerg (unadjusted) H elect (unadjusted)	Good
Chan A, Roder D, & Macharper T. (1988) (English)	Regional, population-based registry, 1981-1983	Australia (South Australia)	5675 “Non-English speaking countries” (includes Italy, Holland, Germany, Vietnam, Greece, Yugoslavia, Philippines, & “other”) vs. 2894 Australian-born.	H overall (all immigrants combined; unadjusted; Variation by groups but not statistically tested) H emerg (all immigrants combined; unadjusted) H elect (all immigrants combined; unadjusted)	Fair
Comas M, Català L, Sala M, et al. (2011) (English)	Local, hospital records, 2006-2007	Spain (Barcelona)	564 Foreign-born (includes women from South America, Asia and North Africa- Morocco, Pakistan and Ecuador most represented) vs. 462 Spanish-born women. Only women who lived in hospital catchment area were included.	ND overall (all immigrants combined; unadjusted)	Fair
Delvaux T, Buekens P, Thoumsin H, Dramaix M, & Collette J. (2003) (English)	Local, research study, 1997-1998	Belgium (Liege)	89 North-African nationality vs. 184 Belgian nationality women. Singleton live births, excluded women with gestational diabetes & malformed babies at birth.	ND overall (unadjusted)	Fair
Diani F, Zanconato G, Foschi F, Turinetto A, & Franchi M. (2003) (English)	Local, hospital records, 1992-2001	Italy (Verona)	1014 “Non-EU women” (including Central Africa, Northern Africa/Middle East, Eastern Europe, Asia, & Latin America) vs. 12,931 Italians.	H overall (all immigrants combined; unadjusted)	Fair
Fedeli U, Alba N, Lisiero M, Zambon F, Avossa F, & Spolaore P. (2010) (English)	Regional, population-based registry, 2006-2007	Italy (Veneto region)	20,332 Regular and Irregular foreign born/migrants vs. 73,098 Italian women.	L overall (unadjusted; when stratified by age for regular migrants ND across strata except ages 30-34 years H)	Fair
Forna F, Jamieson DJ, Sanders D, & Lindsay MK. (2003) (English)	Local, hospital records, 1991-2001	US (Atlanta)	13,465 Africa, Asia, Caribbean, Europe, Central & South America, Middle East & “other” foreign-born vs. 36,439 US born (mostly Black women). Excluded women with no prenatal care.	H & ND overall (unadjusted)	Fair
Gagnon AJ, Dougherty G, Platt RW, et al. (2007) (English) and Gagnon AJ, Van Hulst A, Merry L, et al. (2012) (English) Unpublished when review of literature was conducted, 2007 reference describes original study; 2012 reference is publication of caesarean results (after review of literature was completed)	Local, research study, 2003-2004	Canada (Montreal, Toronto, Vancouver)	1018 “Recently arrived migrants (≤ 5 years)” and defined by UN Macro Region (Europe, Latin America, Africa, West Asia/North Africa, & South-Central Asia), migration status (refugee, asylum seeker, non-refugee immigrant) and length of time (≤ 2 years vs. > 2 years) vs. 2482 Canadian-born. Only low-risk (≤ 35 years, gestational age ≤ 42 weeks) primiparous women.	H & ND overall (unadjusted but restricted to low-risk population) ND emerg (unadjusted but restricted to low-risk population) ND elect (not statistically tested)	Good
Gagnon AJ, Wahoush O, Dougherty G, et al. (2006) (English) Unpublished, 2006 reference describes study	Local, research study, 2006-2009	Canada (Montreal, Toronto, Vancouver)	1025 “Recently arrived migrants” (≤ 5 years) defined by migration status: refugee, asylum seeker, non-refugee immigrant vs. 514 Canadian-born.	ND overall (unadjusted) ND emerg (unadjusted) ND elect (unadjusted)	Fair
Gann P, Nghiem L, & Warner S. (1989) (English)	Local, hospital records, 1981-1987	US (Lowell)	310 Cambodian refugees vs. 110 low income Whites. Only singleton births included. Primary caesareans only.	L overall (unadjusted; ND primips, L multips)	Fair
Gayral-Taminh M, Arnaud C, Parant O, Fournie A, Reme JM, & Grandjean H. (1999) (French)	Local, hospital records, 1988-1994 (excl 1990)	France (Toulouse)	2636 “Black Africa” & Maghreb vs. 3172 French women. Only singleton births included.	H & ND overall (unadjusted; results consistent when stratified by parity) ND during labour caesarean (unadjusted) ND pre-labour caesarean (unadjusted)	Fair
Giani U, Bruzzese D, Pugliese A, Saporito M, & Triassi M. (2011) (Italian, used English abstract)	Regional, population-based registry, 2005	Italy (Campania)	1709 Foreign-born women vs. 28,557 Italian women. Excluded repeat caesareans.	L elect (adjusted)	Good
Harlap S, Kaufman R, Prywes R, Davies AM, Sterk VV, & Weiskopf P. (1971) (English)	Local, research study, 1964-1967	Israel (West Jerusalem)	13,112 Asian, North African, & Western countries (based on birth place) vs. 7635 Israeli women. Only singleton births.	H overall (not statistically tested)	Fair
Hazekamp JT. (1982) (English)	Local, hospital records, 1978	Norway (Oslo)	51 Wives of Pakistani migrant workers vs. 51 Norwegian women matched for age and parity.	ND overall (not statistically tested)	Fair
Helsel D, Petitti DB, & Kunstadter P. (1992) (English)	Regional, population-based registry, 1985-1988	US (Merced & San Joaquin counties, California)	1937 Hmong refugees vs. 3776 non-Hispanic Whites. Mixed marriages excluded. Only singleton births included.	L overall (results consistent across different age and parity strata)	Good
Henry OA, Guaran RL, Petterson CD, & Walstab JE. (1992) (English)	Local, hospital records, 1979-1988	Australia (Melbourne)	1123 Vietnamese (likely refugees) vs. 35,373 Australian born women.	ND overall (adjusted)	Good
Holan S, Vangen S, Hanssen K, & Stray-Pedersen B. (2008) (Norwegian)	Local, research study, 1993-1998	Norway (Oslo)	220 Asian/African women combined (including women from Pakistan, Sri Lanka, India, Vietnam, Morocco, & Somalia) immigrants with diabetes vs. 262 Norwegian women with diabetes. Only singleton births included.	L overall (unclear if statistically significant; results similar when just primips) ND emerg (not statistically tested) ND elect (not statistically tested)	Fair
Howell R. (1989) (English)	Local, hospital records, 1980-1984	Australia (Brisbane)	338 Vietnamese refugees and Filipino women (most married to Caucasian men) vs. 14,790 Australian-born women.	ND overall (unadjusted; ND for primips, L & ND for multips) H emerg (not statistically tested), H (primips) & ND (multips) emerg (unadjusted) L & ND elect (unadjusted but consistent results by parity)	Fair
Ismail KI, Marchocki Z, Brennan DJ, & O’Donoghue K. (2011) (English)	Local, hospital records, 2009	Ireland (Cork)	867 Eastern European women (refers to women from Belarus, Bulgaria, Czech Republic, Hungary, Moldova, Poland, Romania, Russia, Slovakia, & Ukraine); nationality used to categorize; most would be economic migrants. Multiple, breech and elective or pre-labour caesareans were excluded vs. 5550 Irish women.	ND caesareans during labour (unadjusted) L (primips, unadjusted but results consistent when stratified by age) & ND (multips, unadjusted) caesareans during labour	Good
Ismail KI, Marchocki Z, Brennan DJ, & O’Donoghue K. (2010) (English) *Conference abstract*					
Janevic T.(2011)^†^ (English) *Conference abstract*	Local, population-based registry, 1995-2003	US (New York)	511,564 “Foreign-born and ethnicity/region of origin” : North Africa, Sub-Saharan Africa, Africa unspecified, Non-Hispanic Caribbean, Hispanic Caribbean, Mexico, South America, Central America, East Asia, South East Asia/Pacific Islands, & South Central Asian vs. 449,817 US born women. Singleton births only.	M overall (adjusted)	Good
Johnson EB, Reed SD, Hitti J, & Batra M. (2005) (English)	Regional, population-based registry, 1993-2001	US (Washington)	579 Somali immigrants vs. 4837 US-born Whites & Blacks. Only singleton births.	H (compared to US Whites) & ND (compared to US Blacks) (not statistically tested) H (primips) & ND (multips) overall; (adjusted)	Good
Kaminski M. (1975) (French)	Local, research study, 1963-1969	France (Paris)	1795 Migrants from North Africa, South Europe & the Antilles (only women whose husbands were also born outside of France were considered migrants) vs. 5774 French women.	H& ND overall (unadjusted)	Fair
Kingston D, Heaman M, Chalmers B, Kaczorowski J, et al. (2011) ^†^ (English)	National, Population-based survey, 2006	Canada	16,040 “Landed immigrants” (residents) (including North America, Central America, Caribbean/Bermuda, South America, West Europe, East Europe, North Europe, South/unspecified Europe, East Africa, North Africa, West/Central Asia/Middle East/Kurdistan, East Asia, South East Asia/Asia/East Timor, South Asia/Asia non-specified) defined as recent (≤ 5 years) and non-recent arrival (> 5 years) vs. 57,800 Canadian-born. Only singleton births. Weighted numbers.	ND overall (all countries combined & stratified by time since arrival; adjusted; Variation by country but not statistically tested)	Good
Lansakara N, Brown SJ, & Gartland D. (2010) (English)	Local, research study, 2003-2005	Australia (Melbourne)	212 Mothers born overseas of non-English speaking background- needed to be fluent in English to participate; included women from 53 different countries, largest group were from South Asia, Sri Lanka and India vs. 1074 Australian-born mothers. Primiparous women only.	ND overall (all countries combined; unadjusted) ND emerg (all countries combined; unadjusted) ND elect (all countries combined; unadjusted)	Fair
LeRay C, Carayol M, Zeitlin J, Breart G, & Goffinet F. (2006) (English)	National, research study, 2001-2002	France	618 “Origin abroad” (including North Africa, North European, South European, African & Asian) vs. 2797 French. Only low risk primiparous, singleton, cephalic presentation, no induction, Birthweight 2500-4500 g babies, included.	H caesarean during labour (all migrants combined; adjusted; Variation by groups but not statistically tested)	Good
Loew D, & Schrank P. (1966) (German)	Local, hospital records, 1956-1965	Germany (Russelheim)	398 “Foreigners” (including Southern Europe, East Europe & “other”) vs. 6602 German women.	H overall (all foreigners combined; unadjusted)	Fair
Ma J, & Bauman A. (1996) (English)	Regional, population-based registry, 1990-1992	Australia (New South Wales)	64,922 Immigrant women from Europe, Asia, Africa, New Zealand/Oceania, Middle East, America vs. Australian (non-Aboriginal).	M overall (unadjusted)	Fair
Malin M, & Gissler M. (2009) (English)	National, population-based registry, 1999-2001	Finland	6532 Migrants from Latin American/Caribbean, Somali, African, Vietnamese, South East Asian, Iran/Afghan/Iraq, Chinese, South Asian, Middle East, Baltic, Soviet Union, Eastern Europe, Western Europe, & Nordic (only those with resident status) vs. 158,469 Finnish women. Only singleton births.	M overall (unadjusted but results presented by parity)	Fair
Maslovitz S, Kupferminc MJ, Lessing JB, & Many A. (2005) (English)	Local, hospital records, 2001-2002	Israel (Tel Aviv)	721 Non-resident foreign labourers [mostly Eastern Europe, also included women from Africa (Kenya, Nigeria, Ghana) and Asia (Thailand, Philippines, China)] vs. 16,012 Israeli residents.	H overall (all migrants combined; unadjusted) H emerg (all migrants combined; unadjusted) L elect (all migrants combined; unadjusted)	Fair
Merten S, Wyss C, & Ackermann-Liebrich U. (2007) (English)	National, population-based hospital data, 2000-2002	Switzerland	24,284 Migrants based on nationalities from various regions &/or countries of birth: Africa, Latin America, Asia, Balkan/Turkey, EU/USA; Angola, DR Congo, Morocco, Somalia, Tunisia, Brazil, Dominican Republic, Peru, Philippines, Sri Lanka, Thailand, Vietnam, Albania, Bosnia, Croatia, Kosovo, Macedonia, Serbia, Turkey, Austria, France, Germany, Italy, Netherlands, Poland, Portugal, Russia, Spain, UK, & USA vs. 7500 Swiss women.	M overall (adjusted)	Good
Moscioni P, Romagnoli C, Pomili G, & Gilardi G. (1995) (Italian)	Local, hospital records, 1992-1994	Italy (Perugia)	186 “Immigrant/foreign women” vs. 1716 Italian women.	L overall (unadjusted)	Fair
Mossialos E, Allin S, Karras K, & Davaki K. (2005) (English)	Local, hospital records, 2002	Greece (Athens)	181 “Immigrants” (majority from Albania, also included other Balkan countries and India/Philippines/Pakistan) vs. 259 Greek women. Only singleton births.	L overall (all immigrants combined; adjusted)	Good
Oliva GC, Zannella MP, Filidi C, Cavaliere AF, Casarella L, & Mancuso S. (2007) (Italian)	Local, hospital records, 2000-2004	Italy (Rome)	2628 “Foreign women based on nationality” vs. 11,976 Italian women.	L overall (unadjusted) H during labour caesarean (unadjusted) L pre-labour caesarean (unadjusted)	Fair
Panagopoulos P, Tsoukalos G, Economou A, et al. (2005) (English)	Local, hospital records, 2000-2004	Greece (Piraeus)	1990 “Immigrants” (according to nationality) vs. 1081 Greek women.	ND overall (unadjusted)	Fair
Parsons L, Macfarlane AJ, & Golding J. (1993) (English)	National, population-based registry, 1982-1985	UK	Mediterranean, African (excluding East Africa), Bangladeshi, & Pakistani vs. UK women.	M overall (+ variation by parity; unclear if statistically significant)	Fair
Press F, Katz M, Leiberman JR, Shoham I, & Glezerman M. (1993) (English)	Local, hospital records, 1988-1991	Israel (Be’er Sheva)	431 Ethiopian Jewish immigrants vs. 20,047 Israeli Jewish women.	ND overall (unadjusted)	Fair
Richman D, & Dixon S. (1985) (English)	Local, research study, 1980-1981	US (San Diego)	50 Hmong and Cambodian refugees vs. 25 Caucasian (non-Spanish, non-Oriental surnames).	L overall (unadjusted)	Fair
Rio I, Castelló A, Barona C, et al. (2010) (English)	Regional, population-based registry, 2005-2006	Spain (Catalonia & Valencia)	34,746 Latin America, Eastern Europe, & Maghreb vs. 180,633 Spanish women. Only singleton births.	M overall (adjusted for age but not parity)	Fair
Rizzo N, Ciardelli V, Gandolfi-Colleoni G, et al. (2004) (English)	Local, hospital records, 1997-2001	Italy (Bologna)	510 Immigrant women from non-EU countries vs. 510 Western world (Italy, other EU, Australia, Canada, US). Only singleton births.	ND overall (unadjusted) ND emerg (unadjusted) L elect (unadjusted)	Fair
Roman H, Blondel B, Bréart G, & Goffinet F. (2008) (English)	National, population-based survey, 2003	France	585 Africa (excluding North Africa), North Africa, Europe, & “other” nationalities vs. 4658 French women. Only included low risk, singleton births, with no previous caesarean or medical indications for caesarean.	H & ND overall (not statistically tested) H & ND caesareans during labour (adjusted) H & ND caesareans pre-labour (adjusted)	Good
Rudman A, El-Khouri B, & Waldenström U. (2008) ^†^ (English)	National, population-based survey, 2000	Sweden	236 Swedish-speaking Foreign-born vs. 2472 Swedish women.	ND overall (unadjusted) ND emerg (unadjusted) ND elect (unadjusted)	Fair
Rumbaut RG. (1996) (English)	Local, hospital records, 1989-1991	US (San Diego)	1211 “Foreign-born & ethnicity”: defined as White, Asian, Hispanic, & Black; Europe/Canada, Middle East, Indo-Chinese, East Asian, Mexican, Central America & Sub-Saharan Africa vs. 253 US born.	L & ND overall (unadjusted)	Fair
Saurel-Cubizolles M-J, Saucedo M, Drewniak N, Blondel B, & Bouvier-Colle M-H. (2012) (French)	National, Population-based survey, 2010	France	1864 Foreign-born (based on nationality) who could complete the survey in French: European (including Turkey), North-African, Sub-Saharan African, & other; 13% arrived in 2009/2010 and 36% between 2005-2008. vs. 12,125 French women.	H & ND overall (unadjusted) H & ND caesarean during labour (unadjusted) H & ND caesarean pre- labour (unadjusted)	Fair
Saurwein A. (1969) (German)	Local, hospital records, 1964-1968	Germany (Cologne)	297 “Foreigners” from Southern Europe (including Turks, Greeks, Italian, & Spanish women) vs. 7465 German women.	H overall (all immigrants combined; unadjusted)	Fair
Schliemann F, & Schliemann G. (1975) (German)	Local, population-based registry, 1969-1973	Germany (Hamburg)	1217 “Foreigners/Guest workers” from Spain, Italy, Portugal, Yugoslavia, Greece, Turkey & “others” vs. 5112 German women.	H & ND overall (unadjusted)	Fair
Schultze-Naumburg R, & Scholtes G. (1976) (German, used English abstract)	Local, hospital records, 1968-1973	Germany (Berlin)	1941 “Foreign women” vs. 9009 German women.	L overall (not statistically tested)	Fair
Shah D, Tay A, Desai A, Parikh M, Nauta M, & Yoong W. (2011) (English)	Local, hospital records, 2006-2008	UK (North London)	125 First generation Chinese immigrant women (born in China). Economic migrants most likely and mean length of time in UK =3.2 years. vs. 125 British Caucasian women matched for age and parity.	L overall (unadjusted but matched by age and parity)	Good
Shah RR, Ray JG, Taback N, Meffe F, & Glazier R. (2011) (English)	Local, hospital records, 2002-2006	Canada (Toronto)	3672 "Foreign born" based on country and region of birth using World Bank classification: Latin America/Caribbean, Western Europe/USA/Japan/Australia/New Zealand, Eastern Europe/Central Asia, Middle East/North Africa, Sub-Saharan Africa, South Asia, East Asia/Pacific. Asylum seekers examined based on health insurance. < 5years & ≥ 5years also examined. Women with multiple gestation removed. vs. 1435 Canadian-born.	H & ND overall (adjusted)	Good
Shah RR. (2007) (English) *Thesis*					
Sletten K. (2011) (English) *Thesis* unpublished	Local, hospital records, 2009-2010	Norway (Baerum)	803 Immigrants from Latin America, Africa, Asia, Eastern Europe, & Western Europe vs. 1634 Norwegian women. Low risk women (35 weeks or greater gestational age, no diabetes, singleton births) only.	H & ND overall (unadjusted) H & ND emerg (adjusted) L & ND elect (adjusted)	Good
Small R, & Lumley J. (2007) ^†^ (English) *Conference abstract*	Regional, population-based registry, 1999-2007	Australia (Victoria)	70,417 Women from Non-English-speaking countries (only countries w >1000 births were included): Vietnam, China, Former Yugoslavia, Philippines, Lebanon, India, Sri Lanka, Somalia, Ethiopia, Eritrea, Sudan, Turkey, Malaysia, Iraq, Cambodia, Indonesia, Greece, Germany, Poland and Italy vs. 444,175 Australian-born.	M overall (+ variation by parity; not statistically tested)	Fair
Small R, Gagnon A, Gissler M, et al. (2008) (English)	Regional & National, population-based registries, 1997-2004	Australia (New South Wales, Victoria), Belgium (Flanders, Brussels), Canada (Ontario, Quebec), Finland, Norway, Sweden	10,431Somali women vs. 2, 168, 891 receiving-country-born women.	H overall [Belgium- Flanders (primips & multips) & Belgium -Brussels]; H (primips) & ND (multips) overall (Australia- Victoria, Finland, Norway, & Sweden); ND overall [Australia -New South Wales (primips & multips) & Canada] (unadjusted)	Fair
Stray-Pedersen B, & Austveg B. (1996) (Norwegian)	Local, hospital records, 1993	Norway (Oslo)	734 "Immigrants" (including Asia/Africa/South America/East Europe and Turkey) vs. 3188 Norwegians.	H overall (all immigrants combined; unadjusted)	Fair
Teixeira C, Correia S, & Barros H. (2010) (English) *Conference abstract*	Geographical coverage not indicated, research study, years not indicated	Portugal	743 European/North American, African, & South American (based on country of birth) vs. 6692 Portuguese-born. Singleton births only.	H & ND overall (adjusted but variables included not indicated)	Fair
Triantafyllidis G, Tziouva K, Papastefanou I, Samolis S, Katsetos C, & Panagopoulos P. (2010) (English) *Conference abstract*	Local, hospital records, 2007	Greece (Pireaus)	657 "Immigrants" (based on nationality) (mostly Albanians) vs. 304 Greek women. Only term deliveries (gestational age 37-40 weeks).	ND overall (unadjusted)	Fair
Van Enk A, Doornbos HP, & Nordbeck HJ. (1990) (English)	Local, hospital records, 1972-1982	Holland (Amsterdam)	1614 Non-European immigrants defined by ethnic origin: "Blacks" (Surinam & Dutch Antilles), Mediterranean (Turks & Moroccans), Asians [West Indian Asians (Hindustani from Surinam), Chinese & some Indonesians] vs. 6234 Dutch Caucasian.	H & ND overall (unadjusted; results consistent when stratified by parity)	Fair
Van Enk WJ, Gorissen WH, & Van Enk A. (2000) (English)	National, population-based registry, 1990-1993	Holland	5841Migrant teenagers (15-19 years old) defined by ethnic and geographical background: Mediterranean [Turkish & North African (mainly Moroccan)], Black (Surinam & Dutch Antilles), Hindustani (West Indian-Asian from Surinam & Dutch Antilles), Asian (Chinese, Malaysian & Malaccan); non-Dutch European (West & East Europe & American), & "others" (mixed, unknown or other ethnicity) vs. 45,570 Dutch (born in Netherlands and West European origin, includes teenagers and 20-24 yr olds). Singleton, primiparous pregnancies.	M overall (when compared to Dutch teens); L & ND overall (when compared to 20-24 yr old Dutch); (unadjusted but restricted to primips and defined age groups)	Fair
Vangen S, Stoltenberg C, & Schei B. (1996) (English)	Local, hospital records, 1992	Norway (Oslo)	67 Pakistani vs. 70 Norwegian women.	ND overall (unadjusted) ND emerg (unadjusted) ND elect (unadjusted)	Fair
Vangen S, Stoltenberg C, Skrondal A, Magnus P, & Stray-Pedersen B. (2000) (English)	National, population-based registry, 1986-1995	Norway	17,891 Immigrants from Turkey/Morocco, Pakistan, Sri Lanka/India, Vietnam, Philippines, Somalia/Eritrea/Ethiopia, Chile/Brazil vs. 535,600 Norwegians	M overall (adjusted) H & ND emerg (not statistically tested) M elect (not statistically tested)	Good
Vangen S, Stoltenberg C, Johansen RE, Sundby J, & Stray-Pedersen B. (2002) (English)	National, population-based registry, 1986-1998	Norway	1733 Somali vs. 702,192 Norwegians.	H overall (adjusted) H emerg (adjusted) L elect (not statistically tested)	Good
Vangen S, Stray-Pedersen B, Skrondal A, Magnus P, & Stoltenberg C. (2003) (English)	National, population-based registry,1986-1998	Norway	2408 Filipino mother/Filipino father and mixed= Filipino mother/Norwegian father vs. 615,063 Norwegian women.	H overall (adjusted) H emerg (not statistically tested) H & ND elect (adjusted)	Good
Vangen S, Stoltenberg C, Holan S, et al. (2003) (English)	National, population-based registry, 1988-1998	Norway	10,908 "Immigrants" (some had Norwegian citizenship): included countries in North Africa (Morocco, Algeria and Tunisia), South Asia (Pakistan, Sri Lanka, India/Bangladesh) vs. 601,785 Norwegian women. Women with and without diabetes included.	ND overall (all immigrants combined; not statistically tested) ND emerg (all immigrants combined; not statistically tested) ND elect (all immigrants combined; not statistically tested)	Fair
Versi E, Liu KL, Chia P, & Seddon G. (1995) (English)	Local, hospital records, 1987-1991	UK (East London)	6460 Bangladeshi vs. 7592 low-income Caucasian women.	ND overall (unadjusted; results consistent when stratified by parity) ND emerg (not statistically tested) H (multips) & ND (primips) emerg (unadjusted) L (primips & multips) elect (unadjusted)	Fair
Von Katterfeld B, Li J, McNamara B, & Langridge AT. (2011) (English)	Regional, population-based registry, 1998-2006	Australia (West Australia)	59,245 Foreign-born women as per mother's country of birth as declared in the birth register, ten regional categories as per Standard Australia Classification of countries: Oceania, North/West Europe, South/East Europe, North Africa/Middle East, Sub Saharan Africa, South East Asia, North East Asia, South/Central Asia, Americas vs. 149,737Australian-born (non-indigenous).	M overall (not statistically tested) H & ND emerg (unadjusted) M elect (unadjusted)	Fair
Walsh J, Robson M, & Foley M. (2009) (English) *Conference abstract*	Local, hospital records, 2008	Ireland (Dublin)	931 Eastern Europe, Africa, Britain, India, China vs. 2499 Irish women. Only primiparous women, and delivered singleton, term infants.	M caesarean during labour (unclear if statistically significant)	Fair
Walsh J, Mahony R, Armstrong F, Ryan G, O’Herlihy C, & Foley M. (2011) (English)	Local, hospital records, 2008	Ireland (Dublin)	552 Eastern European vs. 2449 Irish women. Primiparous, singleton, term deliveries for women who laboured.	ND caesarean during labourb (adjusted) ND caesarean pre-labour (excluded group from study)	Good
Ryan G, Armstrong F, Walsh J & Foley M. (2010) (English) *Conference abstract*					
Walsh J, Mahony R, McAuliffe F, O'Herlihy C, Robson M, & Foley M. (2009) (English) *Conference abstract*					
Yoong W, Wagley A, Fong C, Chukwuma C, & Nauta M. (2004) (English)	Local, hospital records, 2002	UK (North London)	61 Kosovo-Albanian vs. 61 British Caucasian.	ND overall (unadjusted)	Fair
Yoong W, Kolhe S, Karoshi M, Ullah M, & Nauta M. (2005) (English)	Local, research study, 2002	UK (North London)	69 Somali vs. 69 British Caucasian.	ND overall (unadjusted)	Fair
Zanconato G, Lacovella C, Parazzini F, Bergamini V, & Franchi M. (2011) (English)	Local, hospital records, 2005-2009	Italy (Verona)	2661 “Immigrants" defined as 5 ethnic minority groups based on geographical location: Sub-Saharan Africa, Central & Eastern Europe, Middle East & North Africa, Central and South America, South and East Asia vs. 6365 Italian women; singleton births only.	M overall (adjusted for age but not parity) H & ND caesarean during labour (adjusted for age but not parity) M caesarean pre-labour (not statistically tested)	Fair
Zlot AI, Jackson DJ, & Korenbrot C. (2005) (English)	Local, hospital records, 1994-1998	US (San Diego)	1789 Mexican-born (categorized into acculturation groups by language ability- English, Spanish and bilingual) vs. 313 US born Latina women. Only includes low-risk, low income women.	H & ND (primips) & L & ND (multips) overall; (adjusted)	Good
Zuppa AA, Orchi C, Calabrese V, et al. (2010) (English)	Local, research study, 2005	Italy (Rome)	585 “Immigrants” (includes women from Latin America, East Europe, West Europe, Russia, North America, Asia, Africa) vs. 2334 Italian-born.	L overall (all immigrants combined; unadjusted)	Fair

* For “overall”, “emerg” and “elect” caesarean rates for different migrant groups (defined by country/region of birth, migration status, ethnicity, length of time in new country &/or language ability) compared to non-migrants: H= Higher rate(s); L= Lower rate(s); ND = No Different rate(s); M= Mixed (Higher & Lower rates or Higher, Lower & No Different rates); ‘adjusted’ if at minimum controlled for parity and age. Presented by parity (primiparous = primips; multiparous= multips) &/or age if ‘unadjusted’ and reported.

† Additional data provided.

**Table 2:** Summary of study characteristics

**Characteristic**	**n (number of studies)**	**%**
**Language of publication**	**(n=76)**	
English	62	81.6
French	4	5.3
Italian	3	5.3
Spanish	1	2.6
Norwegian	2	3.9
German	4	1.3
**Publication period**	**(n=76)**	
< 1980	9	11.8
1980-1989	6	7.9
1990-1999	13	17.1
2000-2012	45	59.2
Unpublished	3	3.9
**Data Sources**	**(n=76)**	
Population-based data registries	21	27.6
Population-based hospital data	2	2.6
Population-based surveys	4	5.3
Research study/hospital records	49	64.5
**Variables adjusted**	(n=19)^*^	
Maternal age	19	100
Parity	17	89.5
Education	7	36.8
Gestational age	8	42.1
Marital status	3	15.8
Prenatal care	3	15.8
Social economic status (poverty, income, under-housing, occupation)	4	21.1
Birthweight	9	47.4
Medical complications	2	10.5
Mechanical factors	1	5.3
Previous caesarean	1	5.3
Pregnancy complications (multiple, preterm, fetal stress)	3	15.8
Smoking & substance abuse	1	5.3
Medical indications	2	10.5
Onset of labour	1	5.3
Maternal weight/BMI/diabetes	5	26.3
Assisted reproductive technology	2	10.5
Infant sex	4	21.1
Hospital characteristics (level of care, size of hospital, private facility)	5	26.3
Insurance status	2	10.5
Birth year	1	5.3
Time of day	1	5.3
Location of institution	2	10.5
**Coverage**	**(n=75)**^**†**^	
National	15	19.7
Regional	12	15.8
Local	49	64.5
**Data years**	**(n=75)**^**‡**^	
< 1980	12	12.6
1980–1989	17	17.9
1990–1999	28	29.5
2000-2010	38	40.0
**Number of migrants studied**	**(n=74)**^**Â§**^	
< 1,000	32	43.2
1,000-5,000	23	31.2
> 5,000-10,000	5	6.8
>10,000	14^**^	18.9
**Source regions**	**(n=1,029,454)**^**††**^	
Sub-Saharan Africa	66,163	6.4
North Africa/West Asia	65,689	6.4
Latin America & Caribbean	404,729	39.3
North America/Australia	8855	0.9
East Asia	68,084	6.6
South-East Asia	69,589	6.8
South Asia	70,545	6.9
Asia (unspecified)	46,842	4.6
Oceania (unspecified)	18,005	1.7
East Europe	27,922	2.7
North/West Europe	58,065	5.6
South Europe	16,336	1.6
Unspecified	108,630	10.6
**Receiving countries**	**(n= 76)**^**‡‡**^	
Australia	9	11.1
Belgium	2	2.5
Canada	5	6.2
Finland	2	2.5
France	6	7.4
Germany	4	4.9
Greece	3	3.7
Holland	2	2.5
Ireland	3	3.7
Israel	3	3.7
Italy	9	11.1
Norway	10	12.3
Portugal	1	1.2
Spain	3	3.7
Sweden	3	3.7
Switzerland	1	1.2
UK	6	7.4
US	9	11.1
**Quality of reports**	**(n=76)**	
Good	22	28.9
Fair	54	71.1
**Caesareans**	**(n=64)**^**Â§Â§**^	
Higher	13	20.3
Higher/No different	12	18.8
Lower	9	14.1
Lower/No different	2	3.1
Mixed	8	12.5
No different	20	31.3
**Emergency caesareans**	(n=21)^***^	
Higher	7	33.3
Higher/No different	4	19.0
Lower	0	0.0
Lower/No different	0	0.0
Mixed	0	0.0
No different	10	47.6
**Elective caesareans**	(n=19)^***^	
Higher	2	10.5
Higher/No different	3	15.8
Lower	5	26.3
Lower/No different	2	10.5
Mixed	1	5.3
No different	6	31.6

^*^ Texeira 2010 did not specify variables controlled so excluded; each study may have controlled for more than one variable so total does not equal 19 (or 100%).

^†^ Texeira 2010 did not specify area of coverage; Small 2008 contained regional and national data coverage and so is counted twice.

^‡^ Texeira 2010 did not specify year(s) of data; total adds to more than 74 since a report may have data from more than one time period.

^Â§^ Number of migrants not provided in Giani 2011 and Parsons 1993.

^**^ Kinston 2011 used weighted numbers.

^††^ Total number of migrants studied across reports (overlap accounted for).

^‡‡^ Small 2008 studied migrants in 6 receiving countries.

^Â§Â§^ Giani 2011, Ismail 2011, Le Ray 2006, Walsh, Robson & Foley 2009 and Walsh 2011 are excluded due to lack of data for overall caesarean rates; Studies where rates are not statistically compared are also excluded. Small 2008 was counted as 6 studies since results for 6 receiving countries were reported.

^***^ Studies where rates are not statistically compared are excluded.

Across the 76 studies, data were reported on 1,029,454 migrants (see Table 2). The most common source regions represented were Latin America & Caribbean (39%) followed by ‘origin unspecified’ (11%) and South Asia (7%) (see Table 2). Region and country of origin were the migration indicators most often used to report caesarean rates (57 of 76 studies). “Foreign-born” was used in 16 studies; ethnicity/race/religion/language, length of time and migration status (e.g., “refugees”), were also used in five, three and nine studies respectively.

The receiving countries included Europe (68%), Australia (11%), the US (11%), Canada (6%) and Israel (4%). The primary language of publication was English (82%). Data sources were population-based in 36% of studies, and coverage for the majority (65%) of studies was one city (in most cases one hospital). Data were gathered from 1956 to 2010; 70% from the 1990s or later. More than half of the studies were published from 2000 onward (see Table 2).

### Caesarean Birth Rates: Migrants vs. Non-migrants

Comparison of caesarean rates overall between migrants and non-migrants revealed that in 69% of the studies there were differences in caesarean rates between the two groups (see Table 2). For emergency caesareans, more than half of the studies showed migrants to have higher rates or higher and no different compared to receiving-country-born whereas for elective caesareans there was more variation: 26% showed higher rates or higher and no different; 37% showed lower rates or lower and no different and 5% of studies had mixed results (see Table 2).

### Meta-analyses

#### Sub-Saharan Africa (or Africa unspecified)

**Figure 1:**
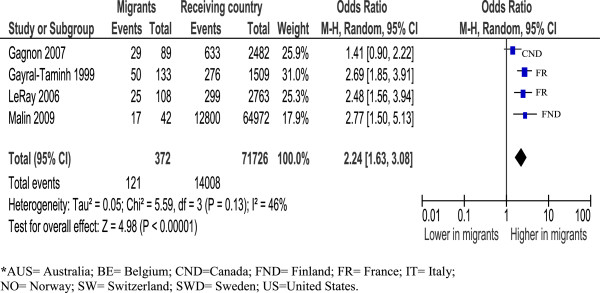
Sub-Saharan Africa primiparous women, overall caesareans.

African migrants had an excess of caesareans compared to receiving-country-born women: France [OR=2.22, (95% CI=1.92, 2.58)] [17,31,94], Australia [OR=1.17 (95% CI=1.11, 1.24)] [34,91]; Canada [OR=1.34 (95% CI=1.08,1.67)] [64,95] and North/West Europe [OR=1.43 (95% CI=1.16, 1.77)] [16,46,82]. US [37,57,81] and Southern European [25,26,88] studies were too heterogeneous to combine, but tended towards higher rates. Analysis by parity showed primiparous African women to be more likely to have a caesarean [OR=2.24 (95% CI=1.63, 3.08)] [31,33,46,64] than non-migrants see Figure 1; multiparous women were also at increased risk [OR=2.02 (95%CI=1.51, 2.71)] [31,46].

Analysis by type of caesarean suggests an excess risk of emergency caesareans for Sub-Saharan African women (all estimates above 1), although results were heterogeneous [17,31,33,64,82,88,91,94]. Risks for elective caesareans varied by receiving-country; three studies in France [17,31,94], one in Italy [88] and another in Australia [91] showed higher risks of a planned caesarean, while two other studies, one in Canada [64] and the other in Norway [82], found African women to have the same and lower risk respectively.

Somali women in North America and Australia were found to have higher risks for a caesarean compared to non-migrant women [OR=1.13 (95% CI=1.02, 1.26)] [32,45]. Significant heterogeneity prevented the calculation of a summary estimate for Somali women in Europe, though the tendency appeared similar to North America/Australia results.

**Figure 2:**
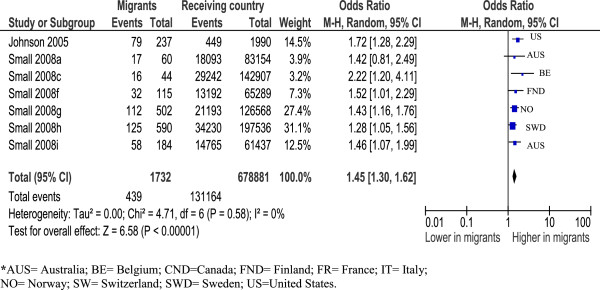
Somali primiparous women, overall caesareans.

Sub-analysis by parity shows primiparous Somali women to have greater risk for caesarean [OR=1.45 (95% CI=1.30, 1.62)] [32,45] (see Figure 2). Multiparous Somali women also appear at increased risk however there was considerable heterogeneity preventing calculation of a summary estimate [32,45]. There were insufficient data for meta-analysis by type of caesarean in Somali women.

#### North Africa (Maghreb)/West Asia (Middle East) (most studies reported results for North-Africa and West Asia combined)

Sub-group analyses by receiving-country showed lower risk of a caesarean in Canada [OR=0.81 (95% CI=0.74, 0.90)] [64,97] and similar risk in France: [OR=1.09 (95% CI=0.95, 1.26)] [17,31,56,73,94]. Analysis for other countries/regions yielded heterogeneous results. With respect to type of caesarean, North African/West Asian migrant women had an elevated risk if the caesarean was an emergency [OR=1.11 (95% CI=1.03, 1.20)] [15,17,31,33,64,88,91,94].

#### Latin America

Differences in caesarean rates between Latin American migrants and non-migrant women depended on receiving-country. Rates were higher in Norway [OR= 2.41 (95%CI=1.79, 3.23)] [15,82], and Canada [OR=1.43(95% CI=1.29, 1.59)] [64,97], whereas in Southern Europe rates were similar [OR=1.03 (95% CI=0.94,1.12)] [26,53,88]. Results in other receiving countries were too few or heterogeneous to combine. Latin American women showed greater risk for an emergency caesarean compared to non-migrant women [OR=1.59(95% CI=1.13, 2.25)] [15,64,82,88]. Results for elective caesareans were too heterogeneous for calculation of a summary estimate.

#### Caribbean (non-Hispanic)

Three older European studies provided data for migrants from former colonized Caribbean states (Holland, UK, France) and combined showed these women were more likely to have caesarean than non-migrants [OR=1.91 (95% CI=1.37, 2.66)] [72,73,78].

#### South Asia

**Figure 3:**
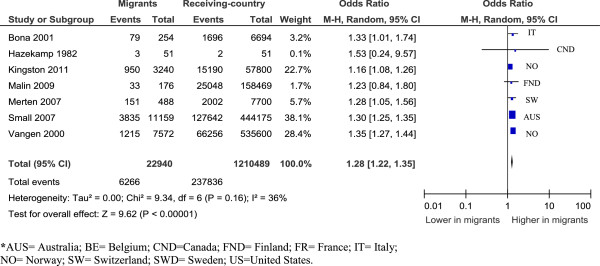
South Asian women, overall caesareans.

Combining studies with similar populations (India, Sri Lanka, unspecified) show these migrant women have an excess of caesareans compared to receiving-country-born women [OR=1.28 (95% CI=1.22, 1.35)] (see Figure 3) [15,16,26,46,58,61,97]. Examined by parity, both primiparous and multiparous women had more caesareans [OR=1.19 (95% CI=1.12, 1.25) and OR=1.39 (95% CI=1.31, 1.47) respectively] [46,61] . Studies for emergency caesareans were too heterogeneous, and there were insufficient data to examine elective caesareans.

#### East Asia (“Far East”)

East Asian women in Southern Europe and the US are less likely to have a caesarean:[OR=0.59, (95% CI=0.47, 0.73)] [25,26] and [OR=0.73 (95% CI=0.71,0.75)] [37,81] respectively, whereas East Asian women in Australia, the UK, Canada and Finland had similar rates to non-migrant women [OR=0.99 (95%CI= 0.95,1.03)] [46,61,91,93,97].

Sub-analysis of three studies of Chinese women [46,61,93] show no difference between migrant and non-migrant women overall [OR=0.97 (95% CI=0.75, 1.25)], for primiparous women [OR=0.93(95%CI=0.86,1.00)] or for multiparous women [OR=1.02 (95% CI=0.95,1.10)] [46,61].

#### South-East Asia

Despite significant heterogeneity, migrant women from Vietnam, Thailand, Cambodia, and Laos had lower risks of caesarean compared to non-migrant women in all nine included studies [15,16,37,46,48,49,51,60,61]. Focusing on Vietnamese women, two older studies found Vietnamese to have caesarean rates lower than Australian-born [OR= 0.87 (95% CI=0.75, 1.00] [49,60]. Similarly more recent studies showed Vietnamese women to have lower rates compared to non-migrant women [OR=0.68 (95% CI=0.66, 0.71)] [16,46,61]. Results were consistent for primiparous [OR=0.78 (95% CI=0.64, 0.94)] and multiparous [OR=0.65(95% CI=0.62, 0.68)] women [46,49,61].

Contrary to other South East Asian women, Filipino women tended towards a greater risk for caesarean birth, although heterogeneity prevented calculation of a summary point estimate [15,16,28,39,49,52,61]. Two Australian studies show multiparous Filipino women to have higher rates [OR=1.19 (95% CI=1.10,1.29)] [49,61].

#### Eastern Europe

Results of studies reporting comparisons of Eastern European women to receiving-country-born are too heterogeneous to calculate a summary point estimate but suggest these women have lower risks for caesarean, with all estimates lower or tending to be lower than one [26,46,53,82,88,97]. Analysis specifically of primiparous women confirmed a reduced risk [OR= 0.52, (95%CI=0.43, 0.63)] [46,87,96]. A sub-analysis of Kosovo women [OR=0.49, (95% CI=0.36, 0.67)] [16,43] and women from Russia/Baltic States living in Europe (Switzerland and Finland) also showed these women to have lower risks [OR=0.75, (95% CI=0.66, 0.85)] [16,46].

#### Southern Europe

Comparisons of women from Italy, Portugal, Spain, and Greece migrating to other parts of Europe or Australia were heterogeneous although the risks of caesarean were consistently greater than that of receiving-country-born women [16,52,56,61,63,68,73].

#### Other

Analyses by other migration indicators (migration status and length of time in receiving-country) were possible for migrants in Canada only. These included an analysis of asylum-seekers who showed no difference in rates compared to Canadian-born [OR=0.93 (95% CI=0.74, 1.17)] [64,66,95]; and migrant women in Canada five years or less who showed a greater risk for caesarean [OR=1.14 (95%CI=1.06, 1.23)] [64,66,97].

### Sensitivity analyses

Meta-analyses with population-based studies confirmed the findings with consistent results for Sub-Saharan African women in France [OR=2.19 (95% CI=1.80,2.67)] [17,94], and South Asian women [OR=1.25 (95% CI=1.17, 1.33)] [16,26,46,61,97]. Sensitivity analyses also support the findings of higher risk of emergency caesareans for North-African/West Asian women [OR=1.09 (95% CI= 1.00,1.19)] [15,17,91,94] and lower risk of caesareans for Eastern European women (heterogeneous but similar estimate). Sensitivity analyses were not informative for Somali and Vietnamese migrants since all studies initially meta-analyzed were population-based, and for Latin American migrants due to an insufficient number of studies.

Summary estimates of adjusted ORs for overall caesarean rates for Sub-Saharan African [OR=1.41 (95%CI=1.19, 1.66)] [16,65,88,95] and Somali women [OR=1.99 (95% CI=1.44, 2.75)] [16,32,41] were consistent in showing higher rates of caesarean compared to non-migrant women. Inconsistent findings were found however when adjusted meta-analyses were performed for South Asian and Latin American migrants; South Asian women still tended towards higher rates but with a wide, non-significant confidence interval [OR=1.10 (95%CI=0.87, 1.38)] [16,95] and Latin American women showed no difference in emergency caesarean rates compared to non-migrant women [OR=1.01 (95% CI=0.62, 1.63)] [82,88]. Adjusted analyses for emergency caesareans in North-African/West Asian women showed a similar point estimate to the unadjusted analyses but with a wide, non-significant confidence interval [OR=1.16 (95% CI=0.86,1.56)] [17,88]. Adjusted results for Eastern European women were too heterogeneous, but did generate a similar estimate to the unadjusted results.

### Assessment for publication bias

Visual inspection of the individual funnel plots for studies reporting overall caesarean rates for Sub Saharan African, Somali, South Asian, Eastern European, Vietnamese, Latin American and North African/West Asian migrants (and emergency caesareans for the two latter) all showed symmetry, suggesting no publication bias, although very small studies were generally lacking.

### Mechanisms/indications for caesareans among migrant women

Table 3 summarizes important risk factors/mechanisms for caesareans in migrants cited in included studies and for each factor the number of studies citing this factor/mechanism. Combined the most commonly reported (in order of frequency) were: language/communication barriers, low social economic status (SES), poor maternal health (e.g., anaemia, STIs, TB, parasitic infections), gestational diabetes/high body mass index (BMI), feto-pelvic disproportion, and lack of prenatal care.

**Table 3:** Potential mechanisms & risk factors involved in caesareans among migrants

Mechanism/Risk factor^*^	**Number of studies**
**Income and social status**	
Low SES (education, income)	15
Higher social status	5
No legal status	2
Poor nutritional status	1
**Social support networks**	
No partner and/or family, friends	4
**Education and literacy**	
High education	3
**Employment and working conditions**	
Low status job	1
**Social environment**	
Poor living conditions	2
**Physical environment**	
Violence, trauma or experiences of abuse	1
**Personal health practices**	
High BMI &/or Gestational diabetes/diabetes	12
Smoking, drug or alcohol abuse	1
**Healthy child development**	
Fetal distress	6
Low birthweight	3
Prematurity	2
**Biology and genetic endowment**	
Illness and other pathologies (e.g., anaemia, hepatitis, TB, malaria, HIV/STIs, parasitic or other infections)	14
Pelvis shape/size (feto-pelvic disproportion)	13
Older age	7
Short stature	4
Pre-eclampsia/hypertension	4
Prolonged labour/failure to progress	3
Post-datism	3
**Health services**	
Language/communication barriers	18
Lack of healthcare including prenatal care	10
Discrimination/racism	1
Hospital environment	1
**Gender**	
Genital cutting	7
**Culture**	
Beliefs/preferences about birth	8
Grand-multiparity	8
Inter-racial marriage (leading to large birthweight babies)	4
Acculturation (adoption of unhealthy lifestyles)	3
Different concepts of health and disease (leading to different health seeking behaviour)	2
Reduced food intake during pregnancy (to have a smaller fetus)	1

^*^ Mechanisms/Risk Factors are organized under 12 determinants of population health (Public Health Agency of Canada (PHAC). Immigration and Health-Exploring the Determinants of Health. Ottawa: Public Health Agency of Canada; 2011).

Evidence to explain the consistently different rates of caesarean between Sub Saharan African, Somali, South Asian, Vietnamese, Eastern European, Latin American and North African/West Asian (emergency caesareans for the two latter) and non-migrant women, was limited. Variables adjusted (see Table 2) or stratified differed across studies and few studies compared indications for caesarean between migrant and non-migrant groups making it difficult to draw firm conclusions regarding explanatory risk factors or mechanisms.

For Somali [15,16,32,41] and Sub-Saharan African [16,17,82,88,95] migrants, adjusted analyses indicate factors other than maternal age, parity, birthweight, or medical complications [e.g., preterm, feto-pelvic disproportion, hypertension, diabetes, BMI] are involved, although it is difficult to know which factors. Prolonged labour due to pelvic shape [17,31,71], genital cutting [15,32,41,45,46,57,88], language barriers [15-17,32,45], poor maternal health (e.g., infectious diseases, anaemia) [31,46,65], a lack of prenatal care [16,33]^,^ and low SES [16,31,32] have all been postulated to be important mechanisms for these women. Gestational diabetes mellitus (GDM) and dystocia were also mentioned as a concern for Sub-Saharan African/Somali women (more than receiving-country-born women) in some studies [32,34,46].

Reports that provided comparisons of medical indications for caesareans between Sub-Saharan African/Somali and non-migrant women show these women to have more caesareans due to fetal distress [31,32] and failed induction [32]. Similarly one study found Sub-Saharan African women who were induced to be more likely to have a caesarean compared to French women who were induced [17], suggesting complications with labour that could be due to any number of different reasons.

Adjusted analyses for South Asian [15] and Latin American (emergency caesareans) [82,88] migrants show age, parity or medical complications (e.g., feto-pelvic disproportion, fetal distress) partially explain excess caesareans in these women, although it is not possible to isolate which of these factors is important. For Latin American women, studies which show consistently higher rates of caesarean irrespective of adjustment of covariates or stratification (e.g., birthweight, hospital type) suggest different contributing factors [15,27,53,65,95]. Seeking social status [55]or cultural preference [15,46,53,95] is one suggested cause; higher rates of pre-eclampsia might also have a role [46]. Stratified analyses by neighbourhood (English-speaking and non-English-speaking) have shown higher rates of caesarean for Latinas compared to US-born when living in non-English-speaking neighbourhoods, leading to the suggestion that communication or cultural barriers might be explanatory mechanisms [27].

For North African/West Asian migrants, hypothesized mechanisms leading to caesarean birth included illness (e.g., hypertension, diabetes) [73], low SES resulting in reduced access to prenatal care [33,73], language barriers [56], and macrosomia [29,70].

Vietnamese [15] and Eastern European [53,87,96] migrants seem to have protective factors that explain their consistently lower rates of caesarean. Proposed protective factors include a preference for a vaginal birth [15,87]; the healthy-immigrant effect (i.e., in which immigrants are healthier than the native population due to immigration selection criteria which excludes individuals with significant health problems) [87,93]; a healthier lifestyle (no smoking, drinking alcohol, or drug abuse; low BMI) [87,96]; young maternal age [87,96]; social support [16] and the use of fewer interventions during labour and birth [87,96]. In Southern Europe (Italy, Spain, Portugal), lower rates among migrants are also thought to be due to a preference for caesareans among non-migrant women [30,50] and because healthcare professionals are more concerned about litigation [25,36,86] from non-migrants.

Filipino migrants were explicitly discussed in a number of studies and hypotheses for their higher caesarean rates included maternal preference [15,39] and feto-pelvic disproportion due to interracial marriage resulting in large birthweight babies [15,28,39,49,52]. More caesareans due to dystocia and feto-pelvic disproportion have been shown, however, this was deemed to not be due to interracial marriage (by comparing Filipino women in mixed marriages to Filipino women in non-mixed marriages) [28,39]. Filipino women who gave birth by caesarean were also found to experience a greater number of medical and obstetric complications (e.g., GDM, anaemia, viral diseases) compared to Australian-born women who delivered by caesarean [28].

## Discussion

Our review of 76 studies comparing the rates of caesarean births between migrant and non-migrant women living in OECD countries, show that women from Sub-Saharan Africa, Somalia and South Asia consistently have an excess of caesareans compared to receiving-country-born women while Eastern European and Vietnamese women have lower overall caesarean rates. North African/West Asian and Latin American women have higher emergency caesarean rates.

The literature provides inadequate empirical evidence to explain differences in caesarean rates observed. Overall it appears that a combination of factors and mechanisms are likely to be involved. The most frequently postulated risk factors for caesarean risk in migrant populations include: language/communication barriers, low SES, poor maternal health, gestational diabetes/high BMI, feto-pelvic disproportion, and lack of prenatal care. There were no studies identified examining determinants of caesareans in migrants specifically. However, gestational diabetes mellitus (GDM) and feto-pelvic disproportion are known to complicate delivery and increase the risk of caesarean [104,105] and these are more common in some migrant women [28,39,106]. Reasons for increased risk of GDM in migrants are unknown but might be associated with a genetic pre-disposition, or physiological response to dietary changes; the involvement of a stress response has also been proposed. Feto-pelvic disproportion might be the result of short stature, a large birthweight baby or childhood malnutrition [107].

A BMI above normal is associated with a number of adverse reproductive outcomes, including GDM, macrosomia, prolonged labour, pre-eclampsia and caesarean [108,109], although the exact mechanisms have not been identified. Excessive weight among childbearing women also varies by ethno-cultural background, with African women representing one of the groups with the highest rates of obesity [94,110].

Inadequate prenatal care is more common among migrants [111]. Language and no health insurance are often cited barriers to healthcare [11,112]. Women who do not receive prenatal care, or who cannot communicate their medical history, may have undiagnosed and untreated medical conditions and they do not benefit from other preventative interventions (e.g., advice about prenatal nutrition) [113]. Medical issues that may not be identified include parasitic infections, anaemia and TB which are known to affect some childbearing migrant women [114-116], although their association with caesarean birth is unclear.

Poverty, unemployment and low social status are significant concerns for migrants [117,118]. Low SES may indirectly be protective against caesarean since women of low SES are less likely to receive care in a private facility and there may be less concern of litigation among healthcare professionals caring for these women. However, low SES may be a barrier to accessing prenatal care, and is known to be independently associated with poorer health. Qualitative studies suggest that a lack of information and/or support during pregnancy and birth due to marginalization, communication barriers, and/or cultural insensitivity (e.g., genital cutting), may result in anxiety, fear and disempowerment [119,120] and lead to unnecessary caesareans.

Confirmation and further understanding of social support as a protective mechanism could have positive implications for reducing caesarean rates among migrants. Additional support (in addition to routine care) including emotional support as well as practical assistance provided during pregnancy has been shown to reduce the risk of caesarean birth for women in socially disadvantaged situations [121]. Continuous support during labour is also associated with a reduced risk of caesarean [122]. This strategy may be particularly beneficial for reducing emergency caesareans, which overall appear to be more common among migrants.

Our findings also suggest that receiving-country is an important variable to consider, particularly for certain groups (e.g., North African, Latin American, East Asian). The degree to which variation in caesarean outcomes across countries represents effects of policies and/or healthcare delivery [123] or other particularities of receiving countries (e.g., cultural factors) versus differences in the migrant populations resettling in each country, remains unknown. More precise definitions of migrants and more complete individual level migration-related data, including source country, length of time in host country, receiving-country language ability (at the time of pregnancy and birth), and migration status (e.g., refugee or economic immigrant) [124] would allow for better interpretation of results particularly since results from Canadian studies have shown that length of residence post-migration (< 5 years) was a significant predictor of more caesarean births. Confirmation of these findings in other OECD countries would be informative.

Research to establish evidence for risk factors associated with caesarean birth in migrants and to deconstruct the pathways (e.g., genetic or physiological, psychological stress, delivery of maternity care) by which they lead to disparities in mode of birth outcomes, is needed. Pathways are likely multi-factorial and complex. Future work using a combination of quantitative and qualitative approaches may be valuable in more fully expounding the processes. Moreover, greater b on differences in caesarean indications/mechanisms would be more informative than simply comparing caesarean rates between migrant and non-migrant women since rates may not be higher, but there may still be disparities in risk factors for caesarean births.

### Strengths and limitations

There are limitations to this review. The web searches, although extensive, did not include all of the government and professional agency websites from all OECD countries. The majority of included studies were rated as ‘fair’ quality for not controlling for confounding or due to some ambiguity in their definitions of the study groups. The heterogeneity due to variation in the migrant populations studied or how source countries were grouped to represent regions, made it challenging to combine data for meta-analysis. US studies were largely missing from meta-analyses due to heterogeneity (for Sub-Saharan African women) or lack of data for the source regions analyzed (for Eastern European, Vietnamese, South Asian, Latin American and North-African/West Asian women). This might be problematic since other systematic reviews comparing the perinatal health of migrants to non-migrants [125,126] had varied outcomes between US and European regions. Nonetheless, a broad range of studies was included and analyzed; the database searches were exhaustive with no time or language limitations and only three reports could not be reviewed due to language. Rigorous methods for reviewing, extracting and analyzing data were applied, optimizing the quality of the results generated. The consistency of the results with population-based data offers confidence in the robustness of the findings.

A major strength of this review is that it is the first of which we are aware to systematically examine hypotheses put forward to explain differences in caesarean rates between migrant and non-migrant women and to assess these in light of the available empirical evidence.

## Conclusion

Sub-Saharan African, Somali, and South Asian migrants consistently have higher caesarean rates while Eastern-European and Vietnamese migrants have lower overall caesarean rates compared to receiving-country-born women. North-African/West Asian and Latin American migrant women have higher emergency caesarean rates. To date there is inadequate empirical evidence to explain observed differences in caesarean rates; more focused research is urgently needed.

## Competing interests

The authors declare that they have no competing interests.

## Authors’ contributions

LM reviewed the literature, extracted data, performed analyses and wrote the first draft of the paper. AG conceived the study, reviewed literature, and critically revised the manuscript for important intellectual content. RS and BB contributed to the design of the study and critically revised the manuscript for important intellectual content. All authors approved of the final version of the paper to be published.

## Pre-publication history

The pre-publication history for this paper can be accessed here:

http://www.biomedcentral.com/1471-2393/13/27/prepub

## Supplementary Material

Additional file 1 Database search strategy. Click here for file

Additional file 2 Websites searched. Click here for file

Additional file 3 UN Macro Regions. Click here for file
