# Supplementary material for: International migration and caesarean birth: a systematic review and meta-analysis
Source: BMC Pregnancy Childbirth. 2013 Jan 30;13:27. doi: 10.1186/1471-2393-13-27 (PMC3621213; doi:10.1186/1471-2393-13-27)
Supplement: Additional file 1 — Database search strategy. [file 1471-2393-13-27-S1.doc]

**Database search strategy**

1.    exp "Emigration and Immigration"/ or exp "Emigrants and Immigrants"/
2.    ethnic groups/
3.    minority groups/
4.    exp Refugees/
5.    (emigra$ or immigra$ or refugee$ or foreigner$ or alien$ or migrat$ or
 migrant$ or premigra$ or racial* or minorit$ or newcomer$ or asylum$).tw.
6.    Ethnology/ or  eh.fs.
7.    (ethno$ or ethnic$).tw.
8.    1 or 2 or 3 or 4 or 5 or 6 or 7

9.   exp "cesarean section"/

10.  (cesarean or caesarean* or c section*

or abdominal deliver* or operative deliver$) .mp.

11.    9 or 10

12.    8 and 11

Limit to humans
