# Supplementary material for: International migration and caesarean birth: a systematic review and meta-analysis
Source: BMC Pregnancy Childbirth. 2013 Jan 30;13:27. doi: 10.1186/1471-2393-13-27 (PMC3621213; doi:10.1186/1471-2393-13-27)
Supplement: Additional file 2 — Websites searched. [file 1471-2393-13-27-S2.doc]

**Websites searched**

- Statistics Canada
- [Canadian socioeconomic database from Statistics Canada](http://www5.statcan.gc.ca/cansim/home-accueil?lang=eng) (CANSIM)
- Canadian Institute for Health Information (CIHI)
- The Society of Obstetricians and Gynaecologists of Canada (SOGC)
- The Association of Women’s Health, Obstetric, and Neonatal Nurses (AWHONN)/ Canadian Association of Perinatal & Women’s Health Nurses (CAPWHN)
- Organization for Economic Co-operation and Development (OECD)
- The Association of Professors of Obstetrics and Gynaecology of Canada (APOG)
- Association des Obstétriciens et Gynécologues du Québec (AOGQ)
- Canadian provincial health websites
- Canadian Association of Midwives (CAM)
- Canadian Foundation for Women’s Health
- Canadian Medical Association (CMA)
- Public Health Agency of Canada (Maternal and Infant Health section)
- Canadian Women’s Health Network (CWHN)
- Canadian Public Health Association (CPHA)
- American College of Nurse-Midwives
- International Cesarean Awareness Network (ICAN)
- Women’s Health Matters
- The Capacity Project/Capacity*Plus*
- National Institute of Child Health & Human Development
- American College of Physicians
- American Congress of Obstetricians & Gynecologists (ACOG)
- Johns Hopkins Bloomberg School for Public Health Information and Knowledge for Optimal Health (INFO) project
- Association of Reproductive Health Professionals (ARHP)
- American Society for Reproductive Medicine (ASRM)
- US Department of Health and Human Services
- Association of Maternal and Child Health Programs (AMCHP)
- Women's eNews
- National Association of Nurse Practitioners in Women's Health
- Harvard Medical School, Center of Excellence in Women's Health
- NSW (New South Wales) Government, Health
- Department of Health, State Government of Victoria
- International Confederation of Midwives (ICM)
- International Center for Research on Women (ICRW)
- Global Health Council
- International Federation of Gynecology and Obstetrics (FIGO)
- WHO, Partnership for Maternal, Newborn, Child Health (PMNCH)
- Institutional Research Repositories: OAIster , eScholarship@McGill, DSPACE
