# Supplementary material for: International migration and caesarean birth: a systematic review and meta-analysis
Source: BMC Pregnancy Childbirth. 2013 Jan 30;13:27. doi: 10.1186/1471-2393-13-27 (PMC3621213; doi:10.1186/1471-2393-13-27)
Supplement: Additional file 3 — UN Macro Regions. [file 1471-2393-13-27-S3.doc]

**UN Macro Regions***

| **Region** | **Countries** |
| --- | --- |
| Sub-Saharan Africa | Countries in Eastern, Western, Southern and Middle Africa: Burundi, Rwanda, Congo, Cameroon, Botswana, Nigeria, Côte d’Ivoire, Somalia |
| North Africa | Morocco, Algeria, Egypt, Tunisia |
| West Asia | Iraq, Saudi Arabia, UAE, Lebanon |
| East Asia | China, Japan |
| South-East Asia | Cambodia, Viet Nam, Thailand |
| South Asia | India, Pakistan, Sri Lanka |
| Latin America | Countries in Southern and Central America: Mexico, El Salvador, Brazil, Chile, Colombia, Peru |
| Caribbean (non-Hispanic) | Suriname, Saint Vincent and the Grenadines, Grenada, Haiti |
| Eastern Europe | Bulgaria, Belarus, Romania, Russian Federation, Poland |
| Southern Europe | Greece, Spain, Italy, Portugal |

***** Table presents selected regions and a brief list of examples of countries; for full details go to: <http://unstats.un.org/unsd/methods/m49/m49regin.htm>
